# European dietitians as key agents of the green transition: An exploratory study of their knowledge, attitudes, practices, and training

**DOI:** 10.3389/fnut.2023.1129052

**Published:** 2023-03-31

**Authors:** Júlia Muñoz-Martínez, Elena Carrillo-Álvarez, Katarzyna Janiszewska

**Affiliations:** ^1^Global Research on Wellbeing (GRoW) Research Group, Blanquerna School of Health Science, Ramon Llull University, Barcelona, Spain; ^2^Pedagogy, Society, and Innovation with ICT support (PSITIC) Research Group, Blanquerna School of Psychology, Education and Sport Sciences, Ramon Llull University, Barcelona, Spain; ^3^The European Federation of the Associations of Dietitians, Naarden, Netherlands

**Keywords:** food systems, sustainable diets, dietitians, planetary diet, sustainable development, Europe

## Abstract

**Introduction:**

How food systems are currently provisioning food to the population is a matter of debate worldwide. Food systems, driven by widespread and increasing adherence to a westernized dietary pattern, are failing to meet people’s basic needs and are draining natural resources. There is a push to make food systems more healthy, fair, and sustainable. To this end, action from all players is needed to meet the international agenda. In this regard, dietitians play a crucial role, as they can provide advice and promote actions that foster the adoption of more sustainable dietary patterns (SDP) as well as the promotion of sustainable food systems. As an emerging requirement in their training, it is crucial to know what dietitians know about SDP as well as their attitudes and current practices in this field in order to strengthen their competences and be key agents for the green transition. For this reason, the aim of the present study is to explore the knowledge, attitudes, practices, and training (KAPT) of European dietitians on SDP by administering an online survey.

**Methods:**

Cross-sectional survey administered between April-August 2021 to dietitians based in the European countries with National Dietetic Associations or Education Associate Members affiliated to the European Federation of Associations of Dietitians (EFAD). Results were analyzed based on European region of professional practice (Northern/Southern/Western/South-East Europe), area of expertise and years of experience.

**Results:**

Responses from 2211 dietitians from 25 countries were received, although the analysis was based on those that responded at least 90% of the survey (*n*=208). European dietitians are lacking training on SDP but are willing to learn more about it. Most dietitians perceive themselves as able to define an SDP, although aspects concerning social and economic sustainability were underestimated. Dietitians concur that barriers exist to the promotion of SDP, such as the lack of updated national food-based dietary guidelines and the absence of support from peers and managers. The country of professional activity seemed to be key to influencing dietitians’ KAPT.

**Discussion:**

These results emphasize the need to strengthen European dietitians’ training in SDP and increase public/private commitment to consider dietitians as key professionals for the transition towards SDP.

## 1. Introduction

Food systems are at the core of debates about sustainability. As a primary link between humans and the planet, they have been established to be paramount to achieving the 172030 Sustainable Development Goals (1). According to the American Dietetic Association (2), “a sustainable food system exists when production, processing, distribution and consumption are integrated and related practices regenerate rather than degrade natural resources, are socially just and accessible, and support the development of local communities and economies.” At the European level, the Farm to Fork strategy, as part of the European Green Deal, was established in 2020 with the aim of accelerating the transition toward more sustainable food systems (3).

Multiple determinants of sustainable food systems have been identified along natural, agricultural, and human systems (4). Nature’s phenomena, such as the climate, nutrient cycling, biodiversity, water cycles, and coastal protection, or natural resources such as the availability and type of land, aquatic systems, forest resources, genetic resources, nutrients, and energy, constitute the foundation for food systems. As such, they intersect with agricultural practices, including the type of production (crops, livestock, forestry, fisheries, and aquaculture) and inputs/outputs (food, feed, plant-based or animal-based commodities/materials, biofuels, etc.). At the human level, economic activities such as processing of nutritious and healthy food, economic development, inclusive and efficient markets, enabling policies and infrastructure, and social services and conditions such as demographic changes, health, nutrition, urbanization, etc. greatly influence food systems.

In this regard, dietary patterns are key downstream elements of the human system. They constitute the demand and, given the availability of resources, drive the agricultural production and use of natural resources (5). Dietary patterns, therefore, also influence economic activities (e.g., food manufacturers want to adapt their products to the preferences of consumers), as well as social aspects (e.g., community activities, or workforce dynamics) (6). Ultimately, dietary patterns impact both human and planetary health (7, 8).

Sustainable Healthy Diets (SHD) are, according to the FAO, *dietary patterns that promote all dimensions of individuals’ health and wellbeing; have low environmental pressure and impact; are accessible, affordable, safe and equitable; and are culturally acceptable* (9). Most of the discourse on sustainable diets focuses on their environmental impact (10–14). However, at least three dimensions have been described: economic, social, and environmental (15). The (human) health dimension is often added to these (16–18) or placed as a dual and interdependent outcome with sustainability (19). The integrity of these dimensions is being threatened by the current westernized dietary patterns, characterized by high consumption of nutritionally poor food products, an excess of animal-source proteins, and a shortage of foods of plant-based origin, and the food system that supports them.

From the environmental point of view, food production, led by consumers’ preferences and driven by weak regulations and large corporations that prioritize short-term public health needs, is accelerating climate change (20). Anthropogenic greenhouse gas emissions (GHGe) (e.g., carbon dioxide, methane, and nitrous oxide) are the root cause of global warming and have been set at the core of the international agenda to achieve CO_2_ neutrality and slow down the rise in temperatures (21). Food systems were recognized in the 2021 UN Food Systems Summit, and in COP26-Glasgow, as key in the fight against climate change. Food systems, agriculture in particular, are estimated to be responsible for up to 34% of GHGe (22), 80% of deforestation, 40% of global land use (23), and 70% of freshwater use (24). However, adaptation strategies on the food production, distribution, and consumption sides are envisioned to mitigate this environmental impact.

Apart from this, a push for a shift toward SHD stems from the burden current diets are posing on global health. The consumption of nutritionally poor diets with low intake of fruits and vegetables and high consumption of processed meat, sugar, and salt has been recognized as the leading cause of disability-adjusted life-years (25). Shifting from current food consumption patterns to diets where especially meat is replaced with other animal sources or plant-based proteins represents a risk reduction of 4% for multiple health outcomes (respiratory disease, cancer, diabetes, and cardiovascular disease combined) and a decrease in GHGe and land use by 24 and 9%, respectively, according to a systematic review of empirical and modeling studies from 2020 (26).

Last but not least, a closer look at the socioeconomic dimension is required to understand the impact of today’s food system. According to the latest estimates provided by FAO in the report “State of Food Security and Nutrition in the World” (27), the current management of food systems is teetering over the socioeconomic principles of SHD. In 2021, 9.8% of the global population was undernourished, and in 2020, almost 3.1 billion people could not afford a healthy diet. The authors highlighted that price inflation and low support from governments to produce nutrient-dense food rather than staples (e.g., rice, sugar, meat) is compromising the purchase of foods such as fruits and vegetables. This is also harming the well-being of small producers, since regulations on food markets mainly favor large corporations (27). This scenario is unlikely to improve as climate change progresses unless strategies that enable greater resilience are applied (28, 29).

For everything mentioned above, it is of great importance to shift toward win-win diets that benefit both the environment and people’s health, pursuing not only a lack of disease but as the full accomplishment of physical, mental and social well-being (30, 31). Dietitians’ role in health promotion through diet is well recognized, but they are being required to also look after the environmental implications of such recommendations. They are envisioned to be key facilitators of the transition toward more sustainable food systems, as they can provide advice and promote actions that foster the adoption of more sustainable dietary patterns (32–35). By providing individual counseling, developing and monitoring food service standards, elaborating food-based dietary guidelines, or implementing other public health measures, dietitians can shape the dietary patterns of a population, thus influencing food systems and impacting its sustainability.

It is therefore fundamental that dietitians be aware of their potential role in fostering SHD and sustainable food systems and possess the competences to adequately promote them by being acquainted with the emerging evidence about what constitutes a sustainable dietary pattern, the factors that favor or hinder sustainable dietary behaviors, and how it changes in different contexts. Since this is an emerging requirement for the dietetics discipline, a systemic change that enable everything mentioned above is needed. To achieve this, it is important to build the foundations by investigating what they already know, what is their attitude toward this topic and their current practices. Previous literature has made this attempt at global (36) and local scale (Australia) (37), but to our knowledge a focus on Europe is still lacking. For this reason, this study aims to explore the knowledge, attitudes, practices, and training (KAPT) of European dietitians on sustainable dietary patterns (SDP).

## 2. Methods

This paper reports on an exploratory cross-sectional study that examines European dietitians’ KAPT on SDP through an online survey, which was available between April and August 2021 to European dietitians from the countries with National Dietetic Associations or Education Associate Members affiliated to the European Federation of Associations of Dietitians (EFAD) (*n* = 28).^1^

We aimed to reach as many respondents as possible from all European countries, areas of expertise and years of experience. Graduated dietitians and final year students of nutrition and dietetics in Europe were eligible. For this research, a convenient and snow-ball sampling strategy was considered the best option given the outreach EFAD has with its network at European level even if it hinders the representativity of the sample. This approach has been used in previous studies with similar characteristics (37, 38). Therefore, dietitians affiliated to the EFAD, including members of the European Specialist Dietetics Networks in Public Health and Obesity, and the European Network of Dietetic Students (ENDietS) were contacted via email to respond the survey. Dissemination was also performed through EFAD communication channels (newsflash, LinkedIn, and Facebook).

Ethical approval to conduct the study was received by the Research Ethical Committee of the Ramon Llull University, and all participants provided written informed consent.

### 2.1. Measures

This research was conducted through an *ad hoc* survey specifically designed for this study using the online platform LimeSurvey. The survey was built following six steps: (1) design the questions to be made in the focus groups based on analysis of the literature and the advice from experienced dietitians; (2) four focus group discussions with European dietitians to explore their KAPT and educational needs on SDP; (3) thematic analysis from step 2; (4) develop the survey, which was structured with five dimensions *(knowledge, educational needs, individuals/consumers/citizens attitudes, preferred educational sources, and barriers/opportunities for being trained in SDP)*; (5) expert validation of the drafted survey with 13 dietitians from multiple backgrounds *(public health, academia, research, food service, and the food industry)*; and (6) pilot administration of the survey with seven dietitians from Germany, Spain, Greece, and the United Kingdom to ensure that the questions were correctly understood and formulated.

The final survey had 52 questions that combined multiple-choice, open-ended, and close-ended answers. The first section of the survey contained questions regarding country of professional activity, area of expertise *(e.g., clinical dietetics, pediatric dietetics, sports dietetic, public health, food service dietetics, students)*, work setting *(e.g., corporate nutrition, education, food industry, hospitals and other public health care facilities, national agencies, private consultancy, public health, and research)* and years of experience *(e.g., 0–4 years, 5–9 years, 10–19 years, >20 years)*. Concerning the questions from the remaining sections, the present paper will display only those questions related to dietitians’ KAPT, which are presented in Table 1. For more details on the answers embedded in each question, see Supplementary Table S1.

**Table 1:** Questions included in the online survey.

Dimension	Question	Type of answer
1. Knowledge	1.1 According to your own perception, please rate from 1 to 4 the following elements depending on their relevance in defining an SDP 1 = low relevance; 4 = high relevance	4-point Likert scale
	1.2 Please rate the following food items from 1 to 10 according to their impact on sustainability 1 = low positive impact; 10 = high positive impact	Closed-ended
	1.3 Please rate the following food characteristics from 1 to 10 according to their impact on sustainability 1 = low positive impact; 10 = high positive impact	Closed-ended
2. Attitude	2.1 On a scale from 1 to 4, how important is the role of dietitians in educating the population in SDP?	4-point Likert scale
	2.2 Would you be able to define what an SDP is?	Closed-ended
	2.3 From a personal point of view, on a scale from 1 to 4, how interested are you in getting to know more about SDP?	4-point Likert scale
3. Practice	3.1 In your opinion, on a scale from 1 to 4, how close are SDP from your way of working?	4-point Likert scale
	3.2 What informational gaps do you encounter to promote/apply SDP?	Multiple choice and open-ended
4. Training	4.1 Have you ever received any kind of training in SDP?	Closed-ended
	4.2 If you need information on SDP, do you know what sources to use?	Closed-ended

Items on the knowledge question: “According to your own perception, please rate from 1 to 4 the following elements depending on their relevance in defining an SDP,” were selected based on the definition of sustainable diets provided by FAO in 2010 and the suggestions made by the expert dietitians. To facilitate the analysis, these items were classified into three categories corresponding to the three dimensions of sustainability as described by the FAO: environmental, economic, or societal.

### 2.2. Data analysis

The data were analyzed using SPSS 28.0.0. Before proceeding with the analysis, the data were cleaned and organized to facilitate the task. Dietitians’ countries of professional activity were categorized according to their respective European region (Northern Europe – N, Western Europe – W, Central-Eastern Europe – CE and Southern Europe – S), following the classification proposed in the European web portal EUR-Lex.^2^ This classification allows to take into consideration some tendencies within regions in terms of culture, climate and agricultural practice. It should be noted that this is not an assumption of the homogeneity of dietitians across the countries within each of the four regions. In this regard, since the distribution of dietitians across areas of expertise was not homogenous, they were grouped into two unique categories to facilitate the analysis. One included food service dietitians and public health nutritionists, referred to in this paper as “Public Health Nutrition.” The other one included clinical dietetics, pediatric dietetics, and sports dietetics and, for the purpose of this article, is categorized as “Clinical Dietetics.” Students also expressed their area of expertise and were allocated according to their answers. Respondents could answer with multiple areas of expertise and work settings, which explains why the sum of percentages in Table 2 is not 100.

**Table 2:** Professional and working characteristics of the sample population (*n* = 208).

	*N*	%
**European region of professional activity**		
Southern Europe	75	36
Western Europe	76	37
Central-Eastern Europe	38	18
Northern Europe	19	9
**Area of expertise (Grouped)**		
Clinical nutrition	114	55
Public health nutrition	92	44
Missing	2	1
**Area of expertise (Ungrouped)**		
Clinical dietetics	123	59
Pediatric dietetics	36	17
Sports dietetics	14	7
Public health nutrition	103	50
Food service dietetics	39	19
Students	20	10
**Work setting**		
Corporate nutrition	15	7
Education	74	36
Food industry	25	12
Hospitals and other public health care facility	66	32
National agency	8	4
Private consultancy	69	33
Public health	45	22
Research	48	23
**Years of experience**		
0–4 years	79	38
5–9 years	38	18
10–19 years	40	19
≥20 years	51	25
**Training in SDP**		
No	157	75
Yes	51	25
Undergraduate	9	4
Post-graduate	17	8
PhD course	4	2
Life-long learning course	12	6
Working group	17	8

Descriptive statistics were performed for those variables reflecting professional characteristics and KAPT. The chi-square test of independence was used to identify professional characteristics that could modulate dietitians’ KAPT. The non-parametric tests Kruskal-Wallis, for evaluating years of experience and country of professional activity, and Mann–Whitney, for evaluating area of expertise, were used to assess differences between groups among continuous variables (questions 1.2 and 1.3).

## 3. Results

A total of 2,211 responses were received, accounting for both partially and fully completed responses. For the present analyses, we included participants that answered at least 90% of the survey. This resulted in the analysis of 208 responses, of which only 4 were partially completed.

From the 28 countries affiliated with EFAD,^3^ answers were received from dietitians working in 25 of these countries. Cyprus, Iceland, and Israel were not represented.^4^ Table 2 shows the survey representation from each European region, and it can be observed that Western countries (*n* = 76; 37%) were the most prevalent ones, followed by those of Southern Europe (*n* = 75; 36%), Central-Eastern Europe (*n* = 38; 18%), and Northern Europe (*n* = 19; 9%).

In reference to the area of expertise, we obtained data mainly from clinical and public health nutritionists and to a lesser extent from pediatric, sports, and food service dietetics. Only two people did not provide an answer to this question, thus resulting in two missing values. Greater homogeneity was observed among dietitians’ work settings, in which education (*n* = 74; 36%), hospitals (*n* = 66; 32%), and private consultancy (*n* = 69; 33%) were the ones that stood out the most, followed by public health (*n* = 45; 22%) and research (*n* = 48; 23%). Working in the food industry (*n* = 25; 12%), within corporate nutrition (*n* = 15; 7%) and in a national agency (*n* = 8; 4%) were the least prevalent work setting.

Concerning years of expertise, there was a higher response rate from dietitians working in the field for less than 5 years (*n* = 79; 37.9%), followed by those more experienced working in the field for more than 20 years (*n* = 51; 24.5%). Those working for 5–10 years, and 10–20 years were 38 (18.2%) and 40 (19.2%), respectively.

Three-quarters of the sample never received training on SDP. Further information on this is provided in the upcoming section named “training.”

### 3.1. Knowledge

Results regarding the perceived degree of relevance of different items in defining SDP, presented in Table 3, showed that around half of the items were considered as completely relevant, as they displayed a median of 4 (maximum punctuation). Items that referred to environmental sustainability reached the median score of 4, i.e., they were more frequently perceived as completely relevant (5 out of 5) in comparison with those involving the economic (1 out of 4) and social dimensions (1 out of 3). Notwithstanding that, the median for the lowest scored items was 3, showing that they were also considered as relevant to the definition of SDP. Three items were scored in better agreement, expressed as an interquartile range (IQR) of 0, and they were related to the environmental dimension (“An SDP ensures health of present and future generations,” “An SDP protects the environment,” “An SDP accounts for the minimum food waste”). As presented in Supplementary Table S2, higher response variability (IQR of 1) was associated with dietitians’ European region of professional activity for two items from the economic dimension (“An SDP ensures fair prices across the food chain” *χ*^2^ = 27.616, *p* < 0.001; “An SDP includes minimally processed foods” *χ*^2^ = 31.993, *p* < 0.001) and two other from the social one (“An SDP is mainly plant-based” *χ*^2^ = 25.334, *p* = 0.003; “An SDP is culturally acceptable,” *χ*^2^ = 24.763, *p* = 0.003). Precisely, more than half of dietitians from Western Europe expressed that ensuring fair prices across the food chain was completely relevant in defining an SDP (51.3%), whereas Central-Eastern dietitians showed the lowest proportion for this response (21.1%). In contrast to this, almost half of Northern dietitians considered this element moderately relevant (47.4%). Regarding food processing, dietitians from Southern Europe tended to consider it as completely relevant to the definition of SDP (57.3%), followed by Central-Eastern (44.7%), Western (43.4%), and Northern dietitians (15.8%). The latter mostly considered this element as being moderately relevant (42.1%). Regarding elements of the social dimension, the highest proportion of Northern dietitians considered SDP as mainly plant-based to be completely relevant (63.2%), in contrast to Central-Eastern dietitians, who gave this answer the least frequency (28.9%) (*χ*^2^ = 25.334, *p* = 0.003). Only within Southern and Western respondents no responses were attributed to the answer “not relevant at all.” Finally, 53.3–55.3% of Southern and Western dietitians, respectively, showed agreement in considering cultural acceptability completely relevant for defining an SDP, whereas Central-Eastern dietitians displayed the lowest percentage for this response (21.1%) (*χ*^2^ = 24.763, *p* = 0.003). Differences between groups were neither observed for further elements nor for other sociodemographic characteristics (area of expertise or years of experience).

**Table 3:** Dietitians’ ratings on the relevance of different elements in defining Sustainable Dietary Patterns.

Related to the environmental dimension	Median	IQR	4* (n; %)	3* (n; %)	2* (n; %)	1* (n; %)
An SDP ensures the health of present and future generations	4	0	169 (81)	28 (13)	11 (5)	0 (0)
An SDP protects the environment	4	0	167 (80)	30 (14)	8 (4)	3 (1)
An SDP ensures the maintenance of soil health	4	1	143 (69)	47 (23)	12 (6)	6 (3)
An SDP includes more ethical and sustainable raising and harvesting of meat (animal welfare)	4	1	135 (65)	53 (25)	14 (7)	6 (3)
An SDP accounts for minimum food waste	4	0	164 (79)	34 (16)	6 (3)	4 (2)
Related to the economic dimension						
An SDP ensures fair prices across the food chain	3	1	84 (40)	82 (39)	32 (15)	10 (5)
An SDP includes local sourcing	4	1	128 (62)	55 (26)	19 (9)	6 (3)
An SDP includes minimally processed foods (e.g., precooked meals, ready to consume fruits/vegetables…)	3	1	96 (46)	63 (30)	41 (20)	8 (4)
An SDP is affordable	3	1	99 (47)	70 (34)	35 (17)	4 (2)
Related to the social dimension						
An SDP is culturally acceptable	3	1	96 (46)	70 (34)	37 (18)	5 (2)
An SDP is mainly plant-based	3	1	99 (48)	63 (30)	43 (21)	3 (1)
An SDP is tasty	4	1	113 (54)	66 (32)	25 (12)	4 (2)

4*, Completely relevant; 3*, Considerably relevant; 2*, Moderately relevant; 1*, Not relevant at all. IQR, interquartile range.

**Figure 1:**
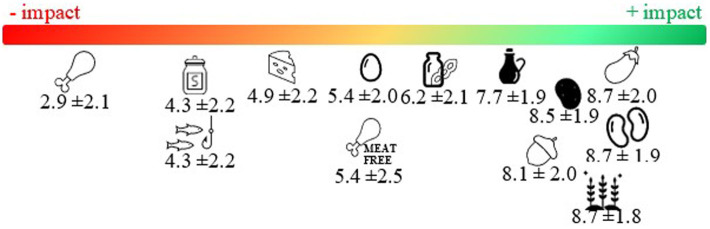
European dietitians mean rating on food items according to their impact on sustainability (1 = more negative impact; 10 = more positive impact).

Dietitians ranked from 1 to 10 the environmental impact of different food items. As expressed in Figure 1, the results showed that the food groups that were perceived to have a more positive impact on the environment were vegetables and fruits, legumes and pulses, whole grains, tubers and roots, and vegetable oils. Rates were generally the same across sociodemographic groups, although significant differences were observed for vegetable oils. Northern dietitians considered vegetable oils as having a more positive impact than the others (Mean 8.5 vs 7.6, *p* < 0.05). Differences were also observed depending on dietitians’ years of expertise. Those who had worked for 5–9 years displayed the lowest rate and those with 10–19 years of experience the highest (Mean 6.4 vs. 8.2, *p* < 0.01). Dairy alternatives, eggs, meat alternatives, dairy, sugar, and fish received neutral punctuation. Differences were also observed depending on the dietitians’ country of professional activity, mainly in regard to fish and dairy. The former received significantly lower punctuation among Western dietitians if compared with the remaining regions (Mean 3.2 vs. 4.8–5.1, *p* < 0.001). Dairy was rated as having a more negative impact also among Western dietitians, in contrast to those from Southern Europe, who rated dairy more positively (Mean 4.1 vs. 5.6, *p* < 0.001). Meat was perceived to be the food group with a greater detrimental impact on sustainability. Greater differences were observed between Western and Central-Eastern dietitians (Mean 2.3 vs. 3.4, respectively, *p* < 0.01). See Tables 4.1, 4.2 for more information.

**Table 4.1:** Differences between dietitians’ mean rating of food items according to their impact on sustainability (1 = more negative impact; 10 = more positive impact) by European region of work.

	Mean	Mean	Mean	Mean	*p*	*p*	*p*	*p*	*p*	*p*
	S	W	N	CE	S vs. W	S vs. N	S vs. CE	W vs. N	W vs. CE	N vs. CE
Meat	3.307 ± 2.604	2.329 ± 1.644	2.211 ± 1.182	3.421 ± 2.088	0.011*	0.094	0.152	0.442	0.002**	0.027*
Meat alternatives	5.947 ± 2.546	5.421 ± 2.424	6.684 ± 2.382	5.500 ± 2.586	0.106	0.004**	0.099	0.031*	0.395	0.065
Fish	4.840 ± 2.260	3.263 ± 1.611	5.158 ± 1.834	4.868 ± 2.559	<0.001**	0.164	0.419	<0.001***	<0.001***	0.149
Eggs	5.773 ± 2.134	4.908 ± 2.060	5.105 ± 1.100	5.579 ± 2.113	0.015*	0.153	0.442	0.361	0.051	0.203
Dairy	5.613 ± 2.199	4.118 ± 2.059	4.474 ± 1.775	5.184 ± 2.335	<0.001***	0.035*	0.156	0.246	0.014*	0.175
Dairy alternatives	6.067 ± 2.101	5.961 ± 2.049	7.211 ± 1.718	6.500 ± 2.037	0.483	0.012*	0.119	0.013*	0.126	0.110
Sugars	4.693 ± 2.193	4.421 ± 2.235	4.842 ± 1.642	4.342 ± 2.317	0.201	0.273	0.188	0.128	0.419	0.119
Legumes and pulses	8.480 ± 2.367	9.013 ± 1.571	9.053 ± 1.224	8.237 ± 1.852	0.155	0.416	0.093	0.333	0.015*	0.128
Wholegrains	8.387 ± 2.295	8.921 ± 1.521	9.263 ± 0.806	8.421 ± 1.703	0.099	0.139	0.282	0.394	0.052	0.081
Tubers and roots	8.267 ± 2.379	8.671 ± 1.739	9.263 ± 0.806	8.526 ± 1.688	0.307	0.100	0.488	0.167	0.328	0.116
Nuts and seeds	8.187 ± 2.216	8.250 ± 1.721	7.684 ± 2.110	8.053 ± 1.888	0.337	0.103	0.223	0.159	0.337	0.270
Vegetable oils	7.680 ± 1.974	7.605 ± 1.841	8.579 ± 1.502	7.605 ± 2.047	0.336	0.035*	0.438	0.019*	0.424	0.039*
Vegetables and fruits	8.507 ± 2.418	8.816 ± 1.874	9.316 ± 1.003	8.605 ± 1.794	0.313	0.157	0.314	0.242	0.188	0.103

*p < 0.05; **p < 0.01; ***p < 0.0001. S, Southern Europe; W, Western Europe; N, Northern Europe; CE, Central-Eastern Europe.

**Table 4.2:** Differences between dietitians’ mean rating of food items according to their impact on sustainability (1 = more negative impact; 10 = more positive impact) by years of experience.

	Mean	Mean	Mean	Mean	*p*	*p*	*p*	*p*	*p*	*p*
	<5 year	5–9 year	10–19 year	>20 year	<5 year vs. 5–9 year	<5 year vs. 10–19 year	<5 year vs. > 20 year	5–9 year vs. 10–19 year	5–9 year vs. > 20 year	10–19 year vs. > 20 year
Meat	2.937 ± 2.215	3.211 ± 2.527	2.550 ± 1.663	2.765 ± 2.055	0.387	0.305	0.312	0.246	0.250	0.479
Meat alternatives	5.443 ± 2.459	5.526 ± 2.648	5.475 ± 2.375	5.098 ± 2.685	0.476	0.481	0.209	0.462	0.231	0.259
Fish	4.177 ± 2.258	3.789 ± 1.933	4.650 ± 2.225	4.588 ± 2.264	0.248	0.147	0.142	0.068	0.063	0.479
Eggs	5.051 ± 2.136	5.079 ± 1.667	5.900 ± 2.193	5.627 ± 2.010	0.490	0.067	0.162	0.096	0.198	0.296
Dairy	4.759 ± 2.392	4.500 ± 1.983	5.525 ± 2.449	4.863 ± 1.876	0.319	0.040*	0.318	0.028*	0.203	0.113
Dairy alternatives	6.291 ± 2.089	5.711 ± 2.117	6.400 ± 2.158	6.314 ± 1.881	0.085	0.497	0.458	0.115	0.120	0.462
Sugars	4.949 ± 2.012	4.132 ± 1.934	4.400 ± 1.972	4.333 ± 2.666	0.023*	0.102	0.013*	0.254	0.496	0.236
Legumes and pulses	8.633 ± 1.909	8.184 ± 2.437	9.075 ± 1.421	8.824 ± 1.873	0.297	0.089	0.148	0.053	0.086	0.363
Wholegrains	8.709 ± 1.741	8.105 ± 2.380	9.025 ± 1.476	8.745 ± 1.753	0.159	0.071	0.230	0.017*	0.062	0.235
Tubers and roots	8.532 ± 1.920	8.079 ± 2.329	8.975 ± 1.493	8.608 ± 1.940	0.174	0.073	0.325	0.073	0.106	0.171
Nuts and seeds	8.139 ± 1.998	7.526 ± 2.357	8.475 ± 1.569	8.333 ± 1.840	0.092	0.276	0.298	0.048*	0.048*	0.462
Vegetable oils	7.722 ± 2.056	6.947 ± 2.092	8.275 ± 1.358	7.863 ± 1.755	0.019*	0.140	0.454	0.003**	0.023*	0.185
Vegetables and fruits	8.911 ± 1.903	8.053 ± 2.427	9.175 ± 1.279	8.529 ± 2.239	0.014*	0.268	0.268	0.007**	0.066	0.137

*p < 0.05; **p < 0.01.

**Figure 2:**
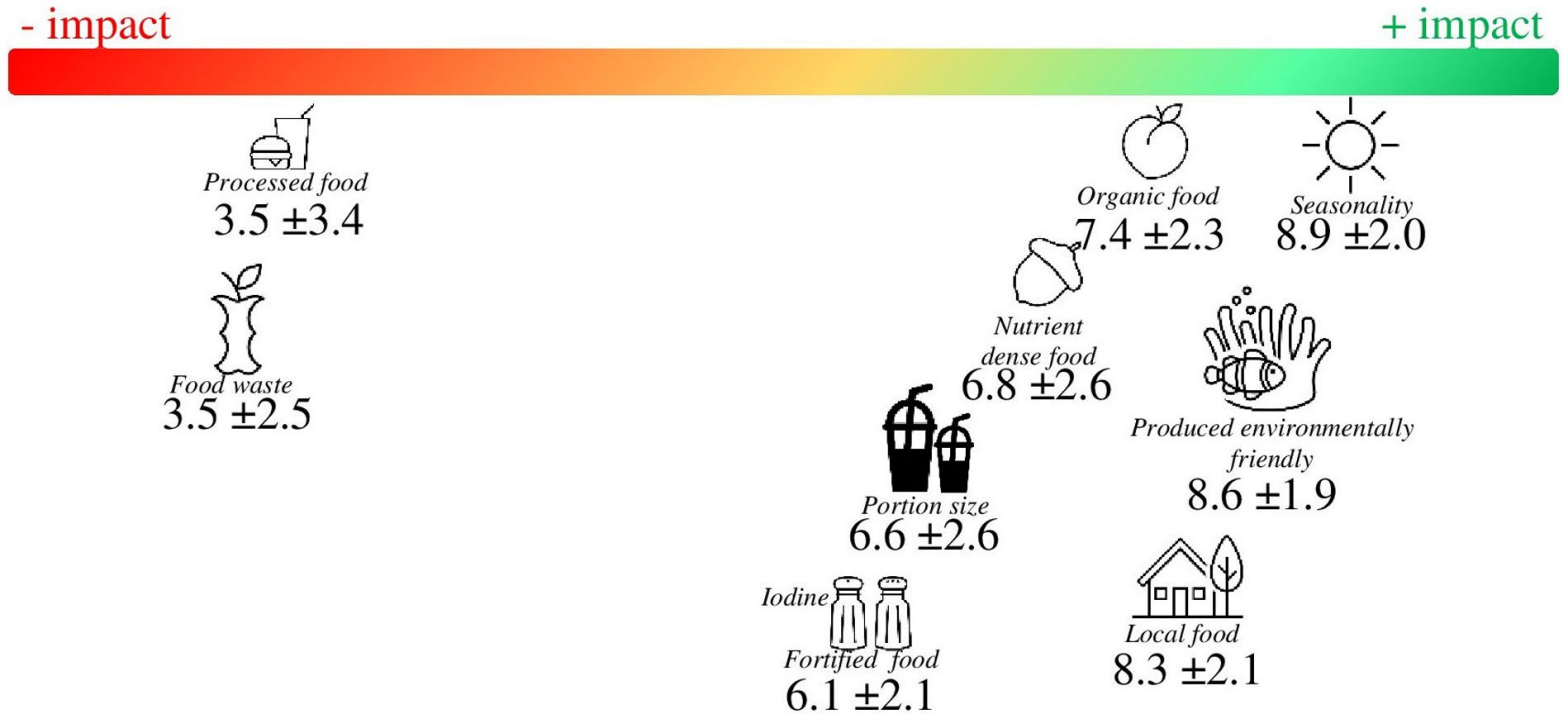
European dietitians mean rating on food characteristics according to their impact on sustainability (1 = more negative impact; 10 = more positive impact).

Regarding the assessment of food characteristics, seasonal foods, those produced in an environmentally friendly manner, and local food, followed by organic food, were rated as being more beneficial to the environment (Mean score: 8.9 ± 2.0, 8.5 ± 1.9, 8.3 ± 2.1, and 7.4 ± 2.3, respectively). To a lesser extent, nutrient-dense food (mean 6.8 ± 2.6), food portion size (6.6 ± 2.6), and micronutrient-fortified foods (mean 6.1 ± 2.1) were perceived as having a more neutral impact on the environment. Food waste (mean 3.5 ± 3.4) and processed food (mean 3.5 ± 2.5) (e.g., precooked meals, ready to consume fruits and vegetables…) were the characteristics perceived as having a more negative impact on the environment (see Figure 2 for more details).

However, as shown in Tables 5.1, 5.2, different ratings were observed depending on the European region and years of experience, mainly for the following food characteristics: processed food, organic food, nutrient-dense food, and micronutrient fortification. No significant differences were observed among dietitians’ areas of expertise.

**Table 5.1:** Differences between dietitians’ mean rating of food characteristics according to their impact on sustainability (1 = more negative impact; 10 = more positive impact) by European region of work.

	Mean	Mean	Mean	Mean	*p*	*p*	*p*	*p*	*p*	*p*
	S	W	N	CE	S vs. W	S vs. N	S vs. CE	W vs. N	W vs. CE	N vs. CE
Processed food	3.440 ± 2.713	3.197 ± 2.173	4.526 ± 2.366	3.711 ± 2.535	0.471	0.010*	0.191	0.009**	0.175	0.066
Local food	8.120 ± 2.573	8.447 ± 1.587	7.737 ± 1.968	8.526 ± 1.913	0.312	0.068	0.347	0.119	0.213	0.050
Seasonal food	8.800 ± 2.433	9.092 ± 1.416	8.474 ± 2.091	8.816 ± 1.943	0.183	0.093	0.239	0.226	0.488	0.239
Organic food	7.240 ± 2.404	7.947 ± 1.959	6.474 ± 1.806	7.289 ± 2.779	0.040*	0.033*	0.326	0.002**	0.164	0.022*
Environmentally friendly produced	8.427 ± 2.249	8.724 ± 1.654	8.368 ± 1.422	8.605 ± 1.939	0.404	0.087	0.418	0.065	0.497	0.082
Food waste	3.853 ± 3.586	3.368 ± 3.174	4.053 ± 3.793	3.053 ± 3.479	0.253	0.327	0.049*	0.488	0.132	0.222
Nutrient dense food	6.653 ± 2.758	6.763 ± 2.383	6.368 ± 3.201	7.289 ± 2.381	0.424	0.374	0.149	0.420	0.115	0.151
Micronutrient fortification	6.293 ± 2.186	5.658 ± 2.004	6.000 ± 2.055	6.632 ± 1.965	0.018*	0.252	0.231	0.252	0.007**	0.129
Food portion size	6.680 ± 2.631	6.263 ± 2.181	6.000 ± 3.496	7.237 ± 2.625	0.103	0.248	0.146	0.452	0.018*	0.086

*p < 0.05; **p < 0.01. S, Southern Europe; W, Western Europe; N, Northern Europe; CE, Central-Eastern Europe.

**Table 5.2:** Differences between dietitians’ mean ratings of food characteristics according to their impact on sustainability (1 = more negative impact; 10 = more positive impact) by years of experience.

	Mean	Mean	Mean	Mean	*p*	*p*	*p*	*p*	*p*	*p*
	<5 year	5–9 year	10–19 year	>20 year	<5 year vs. 5–9 year	<5 year vs. 10–19 year	<5 year vs. > 20 year	5–9 year vs. 10–19 year	5–9 year vs. > 20 year	10–19 year vs. > 20 year
Processed food	3.684 ± 2.233	3.289 ± 2.922	2.800 ± 2.366	3.922 ± 2.489	0.032*	0.005**	0.361	0.275	0.023*	0.004**
Local food	8.367 ± 2.113	7.737 ± 2.490	8.725 ± 1.432	8.196 ± 2.088	0.066	0.351	0.237	0.051	0.216	0.169
Seasonal food	9.038 ± 1.808	8.421 ± 2.708	9.250 ± 1.296	8.686 ± 2.025	0.322	0.391	0.046*	0.261	0.161	0.045*
Organic food	7.418 ± 2.098	6.605 ± 2.824	8.100 ± 1.837	7.569 ± 2.394	0.126	0.057	0.219	0.009**	0.044*	0.215
Environmentally friendly produced	8.810 ± 1.805	8.263 ± 2.321	8.825 ± 1.299	8.196 ± 2.107	0.175	0.237	0.025*	0.420	0.215	0.155
Food waste	3.722 ± 3.679	3.053 ± 3.127	3.300 ± 3.220	3.843 ± 3.455	0.270	0.247	0.226	0.480	0.116	0.103
Nutrient dense food	6.620 ± 2.766	6.026 ± 2.795	6.675 ± 2.314	7.686 ± 2.177	0.112	0.486	0.018*	0.152	0.002**	0.034*
Micronutrient fortification	6.608 ± 2.115	5.474 ± 1.502	5.800 ± 2.198	6.000 ± 2.200	0.003**	0.014*	0.075	0.293	0.087	0.212
Food portion size	6.911 ± 2.266	6.105 ± 2.689	6.600 ± 2.872	6.353 ± 2.704	0.052	0.356	0.167	0.136	0.247	0.314

*p < 0.05; **p < 0.01.

### 3.2. Attitude

Confidence in defining an SDP, assessed through the question “Would you be able to define what an SDP is?,” was observed among 79% (*n* = 165) of dietitians, whereas 21% stated that they would not be able to do so (*n* = 43). Geographical area and years of experience modulated this answer (*p* < 0.05). Up to 86.7% of Southern dietitians and 81.6% from Western Europe were confident in defining SDP, whereas confident dietitians from Central-Eastern and Northern Europe only represented 65.8 and 68.4% of their group, respectively. More experienced dietitians were the most confident in their answers when compared with the remaining groups (94.1% vs. 70–78.5%; *p* = 0.014).

Additionally, it was observed that up to 64% (*n* = 133) of dietitians agreed that their role in promoting SDP was completely relevant, although full agreement was not met since up to 9% (*n* = 19) thought their role was either moderately relevant or not relevant at all. No differences were observed between sociodemographic groups.

Finally, regarding dietitians’ willingness to know more about SDP, although the median score was the highest achievable, agreement was not met. Almost three quarters of the sample were completely interested in getting to know more (*n* = 147; 71%), whereas 13 individuals (6%) were either moderately interested or not interested at all. These results were not influenced by dietitians’ sociodemographic characteristics.

### 3.3. Practice

The application of SDP in practice was generally low. Up to 38% (*n* = 78) of dietitians expressed that their practice was moderately close to SDP principles (punctuation: 2 in a scale of 4) and 36% (*n* = 74) categorized it as moderately close (punctuation: 3 in a scale of 4) (see Table 6 for more details). Differences were observed depending on dietitians’ geographical area of professional activity (*p* < 0.001). Those from Central-Eastern and Northern Europe showed lower rates, since 55.3% from Central-Eastern Europe answered that their practice was “moderately close” (vs 31.6, 34.7, and 36.8% from W, S, and N, respectively) and up to 36.8% of Northern dietitians expressed that their practice was “not close at all” (vs. 4, 10.5, and 18.4% from S, W, and CE, respectively).

**Table 6:** Dietitians’ median and distribution ratings of attitudes and practices on Sustainable Dietary Patterns.

Question	Median	IQR	4* *N* (%)	3* *N* (%)	2* *N* (%)	1* *N* (%)
[Attitude] *“On a scale from 1 to 4, how important is the role of dietitians in educating the population on SDP?”*	4	1	133 (64)	56 (27)	14 (7)	5 (2)
[Attitude] *“From a personal point of view, on a scale from 1 to 4, how interested are you in getting to know more about SDP?”*	4	1	147 (71)	48 (23)	11 (5)	2 (1)
[Practice] “*In your opinion, on a scale from 1 to 4, how close are SDP to your way of working?”*	3	1	31 (15)	74 (36)	78 (38)	25 (12)

4*, Completely relevant; 3*, Considerably relevant; 2*, Moderately relevant; 1*, Not relevant at all. IQR, interquartile range.

**Figure 3:**
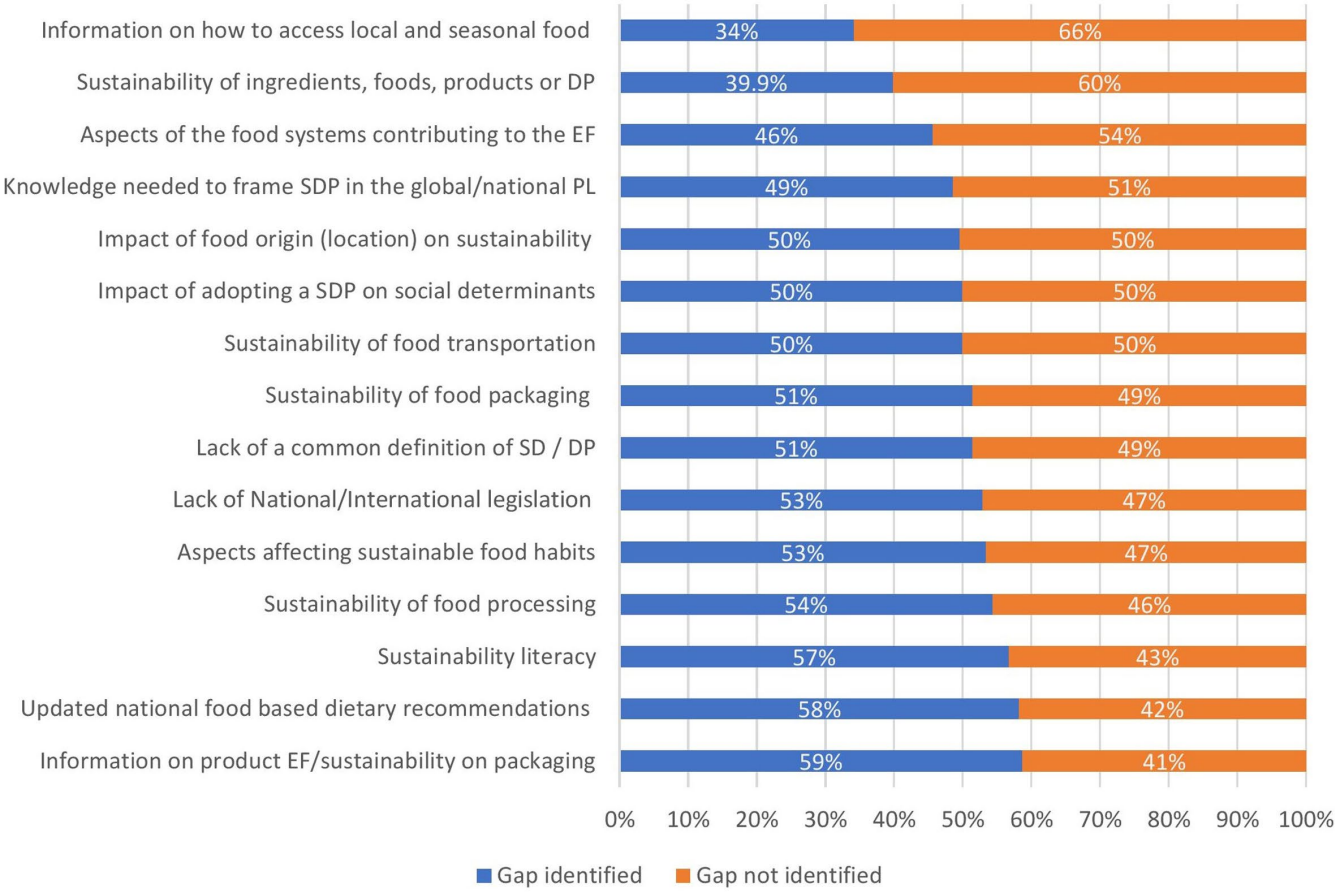
Information gaps identified by dietitians to promote/apply Sustainable Dietary Patterns. DP, Dietary Pattern; EF, Environmental Footprint; PL, Policy Landscape; SD, Sustainable Diets; SDP, Sustainable Dietary Pattern.

Figure 3 displays the results on informational gaps dietitians encountered when applying SDP. Information on a product’s environmental footprint, updated national food-based dietary recommendations, and sustainability literacy were the types of information identified most often (by 59, 58, and 57%, of respondents, respectively). In contrast to this, information on how to access local and seasonal food and the sustainability of ingredients, foods, products, or dietary patterns received fewer votes (34 and 40%, respectively).

As described in Supplementary Tables S3.1, S3.2, significant differences were observed between the responses given by dietitians depending on their geographical region and area of expertise. The lack of national/international guidelines was recognized by roughly half of dietitians from Central-Eastern, Southern, and Western Europe (44.7, 64, and 56.6%, respectively), but only by 10.5% from the North (*p* < 0.001). Similar proportions were observed for information on food packaging sustainability, where Northern dietitians expressed the lowest need for further information compared with those from the remaining regions (21.1% vs. 46.7% S – 57.9% W – 63.2% CE; *p* = 0.011). Dietitians from Central-Eastern Europe had the highest rate of declaring missing information on how to access local and seasonal food (47.4%), followed by those from Southern (38.7%), Western (28.9%) and, ultimately, Northern Europe (10.5%). Regarding food processing, a higher proportion of dietitians from Western (67.1%) and Central-Eastern Europe (57.9%) declared missing information on this, whereas dietitians from Southern and Northern Europe expressing this represented 41.3 and 47.4% of their respective groups.

Strong evidence on the significance of differences depending on area of expertise was observed in sustainability literacy (*p* < 0.001). Up to 69.6% of public health nutritionists expressed missing information on this, almost 50% more than clinical dietitians (46.5%). To a lesser extent, but still significant, small differences were identified regarding information on the sustainability of food origin (*p* = 0.025). In this case, 58.7% of public health nutritionists and 43% of clinical dietitians stated that information was lacking on this. No differences were observed depending on years of expertise.

### 3.4. Training

As indicated in Table 1, only 25% of the sample ever received training on SDP. However, up to 62% (*n* = 128) of dietitians would know what sources of information on sustainability to look for if needed.

In terms of training received, no differences were observed between groups, but there were significant differences when it came to the ability to search for information on SDP (see Table 7). Public health nutritionists were more aware of sources of information about sustainability than clinical dietitians (73.9% vs 52.6%; *p* = 0.002). Years of experience also seemed to influence dietitians’ ability to identify these sources of information, since up to 80.4% of the most experienced ones expressed being able to know what sources to look for, in contrast to lower percentages from the remaining groups ranging from 51.9% (0–4 years) to 65.8% (5–9 years) (*p* = 0.006).

**Table 7:** Dietitians’ awareness of what sources of information to use regarding SDP, stratified by area of expertise, years of experience, and geographical area of work.

	Yes *N* (%)	No *N* (%)	Chi-squared	*p* value
Geographical area				
Central-Eastern	20 (52.6%)	18 (47.4%)	2.976	0.395
Northern	11 (57.9%)	8 (42.1%)		
Southern	45 (60.0%)	30 (40.0%)		
Western	52 (68.4%)	24 (31.6%)		
Area of expertise				
Clinical dietitian	60 (52.6%)	54 (47.4%)	9.801	0.002
Public health nutritionist	68 (73.9%)	24 (26.1%)		
Years of experience				
0–4 years	41 (51.9%)	38 (48.1%)	12.432	0.006
5–9 years	25 (65.8%)	13 (34.2%)		
10–19 years	21 (52.5%)	19 (47.5%)		
≥20 years	41 (80.4%)	10 (19.6%)		

## 4. Discussion

Our study shows that European dietitians are, regardless of their background, undeniably willing to contribute to combating climate change and acknowledge their role in building a more resilient planet. However, almost one quarter of dietitians declare knowing how to define an SDP, and few of them are actually able to apply sustainability in their work settings. The lack of training or structural support such as updated food-based dietary guidelines may contribute to this situation, which worsens depending on dietitians’ country of professional activity. Through the exploration of dietitians’ KAPT toward sustainable diets, this research demonstrates that nutrition and dietetics is an emerging profession in this field and identifies key areas that need to be addressed to enable dietitians to promote sustainable diets effectively.

Regarding knowledge on sustainable diets, our findings show how dietitians identify factors related to environmental sustainability more easily than those related to social and economic sustainability. These results are aligned with a survey conducted among nutrition and dietetic undergraduate students from Australia, where the concepts they were less familiar with were also those related to economic and social sustainability (37). This is not surprising given the fact that the latest literature on food and sustainability is also biased in this way, as it has been stated in previous papers (12, 39, 40). According to a systematic review on indicators for SHD, while 92% of studies included environmental indicators to assess sustainability, only 32% referred to sociocultural parameters (12). In line with this, a report developed by the British Food Agency Standards highlights the absence of studies in the UK evaluating the economic side of sustainability determinants. The complexities that surround the definition of socioeconomic factors from SDP coupled with the publication of reference papers that are solely focused on the environmental aspect of sustainability have contributed to underestimating the socioeconomic aspects of sustainable diets, such as food justice or cultural preferences. However, with the SDG as a guide and the rise of social instability derived from the COVID-19 pandemic and major climatological events, the consideration of the socioeconomic impact of sustainable diets is receiving more attention (27, 29, 41). As a matter of fact, leading organizations such as the EAT Lancet Commission are working toward the publication of reference reports on sustainable diets that include the socioeconomic dimensions (7).

When rating the environmental impact of different food items, a low degree of agreement was observed above all according to dietitians’ geographical region. Moreover, just as it is difficult to determine the socioeconomic impact of SDP, it is difficult to define the environmental one. This is reflected in a systematic review, where the mean environmental impact of food (especially from animal origin) was accompanied by large variations that could be explained by farming methods and conditions (42, 43). Food production, in particular, is crucial to estimate the environmental impact of food, since it accounts for up to 71% of the 34% GHGe coming from agriculture (44). For instance, according to data from the French Environment and Energy Management Agency, 1 kg of lettuce produced in a French heated greenhouse emits 11 kg of CO2eq, whereas 1 kg of in-season lettuce only generates 0.3 kg of CO2eq (42). Apart from this, transportation, food handling, storage, processing, and food trade are also responsible for the environmental impact of food (45). Therefore, how all these stages of the food system are managed will determine the ultimate environmental impact of food.

The proportion of professionals already applying the knowledge and competences linked to this topic in their work setting is low even though dietitians manifest a strong interest in knowing more about it and acknowledge the importance of their profession in promoting SDP. The complexity that surrounds the application of sustainable food systems, the high amount of emerging evidence that prevents dietitians from being up to date, and the lack of agreement on the extent to which dietitians should be part of the change toward sustainable food systems or the absence of support from peers and managers, are some of the obstacles to applying sustainability principles to dietitians work, as identified in a Delphi panel from the International Confederation of Dietetic Associations (36). In order for the dietetics profession to be part of the fight against climate change, it is paramount that they improve their self-efficacy in this matter, and to this end, it is important to strengthen relationships with partners and apply a systemic multi-stakeholder approach (46). Our study shows that European dietitians recognize their role in promoting sustainability, which is in line with other studies conducted worldwide (36). Contradictory results are displayed in studies from the USA, where only 34% acknowledge dietitians’ role in promoting sustainable practices (47). This difference may be explained by the time gap between studies where support from the main institutions has been more evident during the last few years. In recent years, the importance of reducing the environmental impact of food systems, promoting food security, and shifting toward more sustainable diets has been highlighted in numerous leading reports (3, 9, 29), establishing dietitians as professionals in a position to lead change.

In an attempt to highlight dietitians’ relevance in being part of the transition toward SHD, it is important to note that when providing dietary recommendations in light of sustainability, trade-offs can emerge throughout the different stages of food systems (48, 49). From the health perspective, authors have acknowledged that shifting toward more plant-based diets would reduce environmental impact, but bioavailability or intake of key nutrients could be hampered (e.g., proteins, calcium, iron…) (7, 50–52). Also, recommendations could lead to increased intake of processed meat alternatives, which some of them have been recognized as being nutritionally inadequate (53, 54). In this regard, dietitians are trained to design nutritionally adequate diets and are equipped with a strong ability to educate patients and the public, influence allied professionals, and intervene in the different stages of food systems (55). Therefore, to achieve the alignment between sustainability and health, it is fundamental for dietitians to be trained on the basis of sustainability. Our research emphasizes the unprecedented opportunity for universities and other academic organizations to re-assess their curricula and include this topic transversally to their training. In fact, although sustainability may appear as a public health concern, interest in this topic comes from all types of disciplines, thus reflecting that sustainability affects not only public health but also the clinical setting and beyond.

The informational gaps identified in the present paper can be used as a guide to develop these programs. Factors that enable dietitians to be more familiar with SDP key concepts, comprehend current evidence, and apply informed decisions, such as product’s environmental footprint or sustainability literacy, were two of the three main gaps identified by dietitians. Also, the lack of updated FBDG was placed as the second gap dietitians found that impeded their promotion of SDP. Sustainable FBDG are identified as nation-based key tools to enable the transition toward SDP (56). In the European region, 72% of countries (23 out of 32) include sustainability recommendations in their FBDG (57). Nonetheless, this high percentage does not correlate with dietitians perceived lack of updated FBDG. An explanation for this could be that sustainability is included imprecisely and clear messages on how and why sustainability must be embedded in healthy diets are lacking (58). Therefore, sustainability principles are not easily recognized by dietitians. As a matter of fact, only 10% of Northern dietitians identified updated FBDG as a gap, which reflects the efforts made by Northern countries to enable the transition toward SDP. In 2021, the Danish Veterinary and Food Administration updated their FBDG with the slogan “good for health and climate” (59). Furthermore, it is expected that sustainability principles will be incorporated into the Nordic Nutrition Recommendations in 2022, thus aiming to improve diets in Denmark, Finland, Iceland, Norway, and Sweden.^5^

Several socioeconomic factors play a role in how sustainability is applied to dietitians’ practices, including country of professional activity, diet followed, and years of expertise (46). In fact, a scoping review from Guillaumie and colleagues revealed that those having worked for more years expressed fewer intentions to incorporate sustainable nutrition into their practice. However, we found that there was no difference between years of expertise and willingness to apply sustainability to practice and to gain additional knowledge about it. In our case, this survey revealed that the country where dietitians practice their profession was the most influential factor affecting how much sustainable principles were applied to their work. Those whose jobs were based in Western or Central-Eastern Europe were the ones who expressed fewer opportunities to do so. This may be explained by the influence local governance may have on these regions. In the previous paragraphs, the importance of enhancing dietitians’ self-efficacy in promoting sustainability was highlighted, but this is not enough if alliances between key stakeholders are not established, or support from local governance is not preserved (46, 56).

This study entails some limitations. First, the small sample and the unbalanced distribution of dietitians across Europe hampers the drawing of conclusions at the European level. However, this exploratory study provides a first overview of the European situation regarding dietitians’ KAPT on SDP that can inform further studies or actions within the region. Second, governance or training received on SDP are important factors that facilitate dietitians’ application of sustainability principles. However, other sociocultural factors such as the personal dietary patterns of the respondents could also influence their engagement with sustainable practice, as identified in the survey from Hawkins and colleagues (47). Therefore, future research should aim at increasing the sample size and acquiring more information on dietitians’ socioeconomic and cultural characteristics such as the type of diet followed to better understand how their KAPT applies to sustainability principles.

Notwithstanding that, this research is impactful on multiple levels. On the academic side, this is a call for universities and training organizations to deliver courses for dietitians on sustainability and include this in undergraduate degrees. The differences observed across European regions underline the importance of strengthening the commitment of public authorities to develop FBDGs that are up to date with sustainability principles, hence facilitating the promotion of SD among dietitians. Finally, our research can also be used by private companies engaged with nutrition and sustainability to recognize dietitians as professionals than can assist them meet their business goals.

## Data availability statement

The raw data supporting the conclusions of this article will be made available by the authors, without undue reservation.

## Ethics statement

The studies involving human participants were reviewed and approved by the Research Ethical Committee of the Ramon Llull University. The patients/participants provided their written informed consent to participate in this study.

## Author contributions

JM-M and EC-Á involved in the conceiving of the study, organized the database, performed the statistical analyses, and wrote the manuscript. All authors contributed to the manuscript revision and approved the submitted version.

## Funding

This research has been conducted with the support of an unrestricted educational grant from Nestle and Danone. The funder was not involved in the study design, collection, analysis, interpretation of data, the writing of this article, or the decision to submit it for publication.

## Conflict of interest

KJ declares that the EFAD received an unrestricted educational grant from Nestle and Danone to establish a Sustainability in Dietetics course, and her role in EFAD during the preparation of the manuscript has been partially covered by this grant.

The remaining authors declare that the research was conducted in the absence of any commercial or financial relationships that could be construed as a potential conflict of interest.

## Publisher’s note

All claims expressed in this article are solely those of the authors and do not necessarily represent those of their affiliated organizations, or those of the publisher, the editors and the reviewers. Any product that may be evaluated in this article, or claim that may be made by its manufacturer, is not guaranteed or endorsed by the publisher.

## Footnotes

^1^ https://www.efad.org/membership/full-and-affiliate-members/

https://www.efad.org/education-associate-members/

^2^ https://eur-lex.europa.eu/browse/eurovoc.html?params=72,7206,911#arrow_911

^3^ https://www.efad.org/membership/full-and-affiliate-members/

https://www.efad.org/education-associate-members/

^4^ https://eur-lex.europa.eu/browse/eurovoc.html?params=72,7206,911#arrow_911

^5^ https://www.who.int/europe/news/item/20-10-2021-making-diets-environmentally-friendly-nordic-countries-lead-the-way
