# Supplementary material for: European dietitians as key agents of the green transition: An exploratory study of their knowledge, attitudes, practices, and training
Source: Front Nutr. 2023 Mar 31;10:1129052. doi: 10.3389/fnut.2023.1129052 (PMC10102641; doi:10.3389/fnut.2023.1129052)
Supplement: Supplementary file 1 [file Data_Sheet_1.docx]

| **TABLE S1** Questions included in the online survey | | | |
| --- | --- | --- | --- |
| **DIMENSION** | **QUESTION** | **TYPE OF ANSWER** | **ANSWERS** |
| 1. Knowledge | 1.1 According to your own perception, please rate from 1 to 4 the following elements depending on their relevance in defining a SDP  *1= low relevance; 4= high relevance* | 4 points Likert-scale | Environmental elements*   - A SDP ensures health of present and future generations - A SDP protects the environment - A SDP ensures the maintenance of soil health - A SDP includes a more ethical and sustainable raising and harvesting of meat (animal welfare) - A SDP accounts for the minimum food waste   Economic elements*   - A SDP ensures fair prices across the food chain - A SDP includes local sourcing - A SDP includes minimally processed foods (i.e. precooked meals, ready to consume fruits/vegetables… - A SDP is affordable   Social elements*   - A SDP is mainly plant-based - A SDP is culturally acceptable - A SDP is tasty |
|  | 1.2 Please rate the following food items from 1 to 10 according to their impact on sustainability  *1= low positive impact; 10= high positive impact* | Closed-ended | - Meat - Meat alternatives - Fish - Eggs - Dairy - Dairy alternatives - Sugars - Legumes and pulses - Whole grains - Tubers and roots - Nuts and seeds - Vegetable oils - Vegetables and fruits |
|  | 1.3 Please rate the following food characteristics from 1 to 10 according to their impact on sustainability  *1= low positive impact; 10= high positive impact* | Closed-ended | - Processed food (p.e. precooked meals, ready to consume fruits/vegetables…) - Local food - Seasonal foods - Organic foods - Environmentally friendly produced food - Food waste - Nutrient dense food - Micronutrient fortified foods - Food portion sizes |
| 1. Attitude | 2.1 On a scale from 1 to 4, how important is the role of dietitians in educating the population in SDP? | 4 points Likert-scale | 1 = not important at all  2 = moderately important  3 = considerably important  4 = completely important |
|  | 2.2 Would you be able to define what a SDP is? | Closed-ended | - Yes - No |
|  | 2.3 From a personal point of view, on a scale from 1 to 4, how interested are you in getting to know more about SDP? | 4 points Likert-scale | 1 = not interested at all  2 = moderately interested  3 = considerably interested  4 = completely interested |
| 1. Practice | 3.1 In your opinion, on a scale from 1 to 4, how close are SDP from your way of working? | 4 points Likert-scale | 1 = not close at all  2 = moderately close  3 = considerably close  4 = completely close |
|  | 3.2 What informational gaps do you encounter to promote/apply SDP? | Multiple choice and open-ended | - Lack of National/International legislation - Updated national food based dietary recommendations to include sustainability aspect - Knowledge (gap) needed to frame SDP in the global and national policy landscape - Lack of a common definition of sustainable diet/ dietary pattern - Information on product environmental footprint / sustainability on packaging (e.g. nutritional / environmental scores and sustainability labelling) - Sustainability literacy (Clear understanding of gas emissions, land/water use, biodiversity) - Sustainability of food processing - Sustainability of food packaging - Sustainability of food transportation - What aspects of the food systems contribute more or less to the environmental footprint (carbon emissions, land use, water use…) - Impact of food origin (location) on sustainability - Information on how to access local and seasonal food - Sustainability of specific ingredients, foods, products or dietary patterns - Impact of adopting a SDP on social determinants (affordability, acceptability…) - Aspects affecting sustainable food habits (schools, medical stuff, parental support, public health experts, etc) - None of the above - Other |
| 1. Training | 4.1 Have you ever received any kind of training in SDP? | Closed-ended | - Yes - No |
|  | 4.2 If you need information on SDP, do you know what sources to use? | Closed-ended | - Yes - No |
| **The classification of the different sustainability elements has followed the three categories proposed by FAO when defining sustainable diets* (FAO, 2018a)*.* | | | |

| **TABLE S2** Dietitians rating on the importance of different elements in defining Sustainable Dietary Patterns depending European geographical area of work | | | | | | | |
| --- | --- | --- | --- | --- | --- | --- | --- |
|  | **European region** | **Not relevant at all** | **Moderately relevant** | **Considerably relevant** | **Completely relevant** | **Chi-squared** | **p value** |
| **Ensures health of present and future generations** | Central-Eastern | 0 | 3 (7.8%) | 5 (13.1%) | 30 (78.9%) | 2.607 | 0.856 |
|  | Northern | 0 | 0 (0%) | 3 (15.8%) | 16 (84.2%) |  |  |
|  | Southern | 0 | 4 (5.3%) | 12 (16.0%) | 59 (78.7%) |  |  |
|  | Western | 0 | 4 (5.3%) | 8 (10.5%) | 64 (84.2%) |  |  |
| **Protects the environment** | Central-Eastern | 0 (0%) | 2 (5.3%) | 8 (21.0%) | 28 (73.7%) | 9.075 | 0.430 |
|  | Northern | 1 (5.3%) | 1 (5.3%) | 2 (10.5%) | 15 (78.9%) |  |  |
|  | Southern | 0 (0%) | 4 (5.3%) | 12 (16.0%) | 59 (78.7%) |  |  |
|  | Western | 2 (2.6%) | 1 (1.3%) | 8 (10.5%) | 65 (85.5%) |  |  |
| **Ensures the maintenance of soil health** | Central-Eastern | 0 (0%) | 5 (13.1%) | 10 (26.3%) | 23 (60.5%) | 15.692 | 0.074 |
|  | Northern | 1 (5.2%) | 2 (10.5%) | 4 (21.0%) | 12 (63.2%) |  |  |
|  | Southern | 1 (1.3%) | 5 (6.7%) | 20 (26.7%) | 49 (65.3%) |  |  |
|  | Western | 4 (5.3%) | 0 (0%) | 13 (17.1%) | 59 (77.6%) |  |  |
| **Includes a more ethical and sustainable raising and harvesting of meat** | Central-Eastern | 0 (0%) | 1 (2.6%) | 12 (31.6%) | 25 (65.8%) | 16.278 | 0.061 |
|  | Northern | 1 (5.3%) | 3 (15.8%) | 7 (36.8%) | 8 (42.1%) |  |  |
|  | Southern | 0 (0%) | 6 (8%) | 14 (18.7%) | 55 (73.3%) |  |  |
|  | Western | 5 (6.6%) | 4 (5.3%) | 20 (26.3%) | 47 (61.8%) |  |  |
| **Ensures fair prices across the food chain** | Central-Eastern | 2 (5.3%) | 7 (18.4%) | 21 (55.3%) | 8 (21.1%) | 27.616 | 0.001 |
|  | Northern | 1 (5.3%) | 9 (47.4%) | 3 (15.8%) | 6 (31.6%) |  |  |
|  | Southern | 3 (4%) | 10 (13.3%) | 31 (41.3%) | 31 (41.3%) |  |  |
|  | Western | 4 (5.3%) | 6 (7.9%) | 27 (35.5%) | 39 (51.3%) |  |  |
| **Includes local sourcing** | Central-Eastern | 0 (0%) | 3 (7.9%) | 12 (31.6%) | 23 (60.5%) | 15.163 | 0.087 |
|  | Northern | 2 (10.5%) | 4 (21.1%) | 3 (15.8%) | 10 (52.6%) |  |  |
|  | Southern | 0 (0%) | 6 (8%) | 17 (22.7%) | 52 (69.3%) |  |  |
|  | Western | 4 (5.3%) | 6 (7.9%) | 23 (30.3%) | 43 (56.6%) |  |  |
| **Is mainly plant-based** | Central-Eastern | 1 (2.6%) | 9 (23.7%) | 17 (44.7%) | 11 (28.9%) | 25.334 | 0.003 |
|  | Northern | 2 (10.5%) | 3 (15.8%) | 2 (10.5%) | 12 (63.2%) |  |  |
|  | Southern | 0 (0%) | 13 (17.3%) | 26 (34.7%) | 36 (48%) |  |  |
|  | Western | 0 (0%) | 18 (23.7%) | 18 (23.7%) | 40 (52.6%) |  |  |
| **Accounts for the minimum food waste** | Central-Eastern | 0 (0%) | 0 (0%) | 5 (13.2%) | 33 (86.8%) | 9.686 | 0.377 |
|  | Northern | 1 (5.3%) | 2 (10.5%) | 2 (10.5%) | 14 (73.7%) |  |  |
|  | Southern | 1 (1.3%) | 3 (4%) | 12 (16%) | 59 (78.7%) |  |  |
|  | Western | 2 (2.6%) | 1 (1.3%) | 15 (19.7%) | 58 (76.3%) |  |  |
| **Includes minimally processed foods (p.e. prcooked meals, ready to consume fruits/vegetables…)** | Central-Eastern | 0 (0%) | 10 (26.3%) | 11 (28.9%) | 17 (44.7%) | 31.993 | < 0.001 |
|  | Northern | 4 (21.1%) | 8 (42.1%) | 4 (21.1%) | 3 (15.8%) |  |  |
|  | Southern | 2 (2.7%) | 9 (12%) | 21 (28%) | 43 (57.3%) |  |  |
|  | Western | 2 (2.6%) | 14 (18.4%) | 27 (35.5%) | 33 (43.4%) |  |  |
| **Is culturally acceptable** | Central-Eastern | 0 (0%) | 9 (23.7%) | 21 (55.3%) | 8 (21.1%) | 24.763 | 0.003 |
|  | Northern | 2 (10.5%) | 2 (10.5%) | 9 (47.7%) | 6 (31.6%) |  |  |
|  | Southern | 2 (2.7%) | 13 (17.3%) | 20 (26.7%) | 40 (53.3%) |  |  |
|  | Western | 1 (1.3%) | 13 (17.1%) | 20 (26.3%) | 42 (55.3%) |  |  |
| **Is affordable** | Central-Eastern | 0 (0%) | 10 (26.3%) | 15 (39.5%) | 13 (34.1%) | 8.283 | 0.506 |
|  | Northern | 0 (0%) | 2 (10.5%) | 8 (42.1%) | 9 (47.4%) |  |  |
|  | Southern | 2 (2.7%) | 12 (16.0%) | 26 (34.7%) | 35 (46.7%) |  |  |
|  | Western | 2 (2.6%) | 11 (14.5%) | 21 (27.6%) | 42 (55.3%) |  |  |
| **Is tasty** | Central-Eastern | 0 (0%) | 5 (13.2%) | 18 (47.4%) | 15 (39.5%) | 14.405 | 0.109 |
|  | Northern | 1 (5.3%) | 4 (21.1%) | 5 (26.3%) | 9 (47.4%) |  |  |
|  | Southern | 0 (0%) | 10 (13.3%) | 24 (32.0%) | 41 (54.7%) |  |  |
|  | Western | 3 (3.9%) | 6 (7.9%) | 19 (25.0%) | 48 (63.2%) |  |  |

| **TABLE S3.1** Informational gaps identified by dietitians depending on geographical area of professional activity | | | | | | |
| --- | --- | --- | --- | --- | --- | --- |
|  | **Central-Eastern** | **Northern** | **Southern** | **Western** | **Chi-squared** | **p value** |
| **2_Lack of National/International legislation** | | | | | | |
| Yes -N (%) | 17 (44.7%) | 2 (10.5%) | 48 (64.0%) | 43 (56.6%) | 18.829 | < 0.001 |
| No - N (%) | 21 (55.3%) | 17 (89.5%) | 27 (36.0%) | 33 (43.4%) |  |  |
| **3_Updated national food based dietary recommendations to include sustainability aspect** | | | | | | |
| Yes -N (%) | 25 (65.8%) | 10 (52.6%) | 45 (60.0%) | 41 (53.9%) | 1.806 | 0.614 |
| No - N (%) | 13 (34.2%) | 9 (47.4%) | 30 (40.0%) | 35 (46.1%) |  |  |
| **4_Knowledge (gap) needed to frame SDP in the global and national policy landscape** | | | | | | |
| Yes -N (%) | 22 (57.9%) | 6 (31.6%) | 38 (50.7%) | 35 (46.1%) | 3.843 | 0.279 |
| No - N (%) | 16 (42.1%) | 13 (68.4%) | 37 (49.3%) | 41 (53.9%) |  |  |
| **5_Lack of a common definition of sustainable diet/ dietary pattern** | | | | | | |
| Yes -N (%) | 21 (55.3%) | 11 (57.9%) | 37 (49.3%) | 38 (50.0%) | 0.736 | 0.865 |
| No - N (%) | 17 (44.7%) | 8 (42.1%) | 38 (50.7%) | 38 (50.0%) |  |  |
| **6_Information on product environmental footprint / sustainability on packaging (e.g. nutritional / environmental scores and sustainability labelling)** | | | | | | |
| Yes -N (%) | 21 (55.3%) | 11 (57.9%) | 44 (58.7%) | 46 (60.5%) | 0.295 | 0.961 |
| No - N (%) | 17 (44.7%) | 8 (42.1%) | 31 (41.3%) | 30 (39.5%) |  |  |
| **7_Sustainability literacy (Clear understanding of gas emissions, land/water use, biodiversity)** | | | | | | |
| Yes -N (%) | 18 (47.4%) | 11 (57.9%) | 43 (57.3%) | 46 (60.5%) | 1.825 | 0.610 |
| No - N (%) | 20 (52.6%) | 8 (42.1%) | 32( 42.7%) | 30 (39.5%) |  |  |
| **8_Sustainability of food processing** | | | | | | |
| Yes -N (%) | 22 (57.9%) | 9 (47.4%) | 31 (41.3%) | 51 (67.1%) | 10.670 | 0.014 |
| No - N (%) | 16 (42.1%) | 10 (52.6%) | 44 (58.7%) | 25 (32.9%) |  |  |
| **9_Sustainability of food packaging** | | | | | | |
| Yes -N (%) | 24 (63.2%) | 4 (21.1%) | 35 (46.7%) | 44 (57.9%) | 11.064 | 0.011 |
| No - N (%) | 14 (36.8%) | 15 (78.9%) | 40 (53.3%) | 32 (42.1%) |  |  |
| **10_Sustainability of food transportation** | | | | | | |
| Yes -N (%) | 21 (55.3%) | 5 (26.3%) | 36 (48.0%) | 42 (55.3%) | 5.646 | 0.130 |
| No - N (%) | 17 (44.7%) | 14 (73.7%) | 39 (52.0%) | 34 (44.7%) |  |  |
| **11_What aspects of the food systems contribute more or less to the environmental footprint (carbon emissions, land use, water use…)** | | | | | | |
| Yes -N (%) | 11 (28.9%) | 8 (42.1%) | 35 (46.7%) | 41 (53.9%) | 6.509 | 0.089 |
| No - N (%) | 27 (71.1%) | 11 (57.9%) | 40 (53.3%) | 35 (46.1%) |  |  |
| **12_Impact of food origin (location) on sustainability** | | | | | | |
| Yes -N (%) | 13 (34.2%) | 9 (47.4%) | 43 (57.3%) | 38 (50.0%) | 5.437 | 0.142 |
| No - N (%) | 25 (65.8%) | 10 (52.6%) | 32 (42.7%) | 38 (50.0%) |  |  |
| **13_Information on how to access local and seasonal food** | | | | | |  |
| Yes -N (%) | 18 (47.4%) | 2 (10.5%) | 29 (38.7%) | 22 (28.9%) | 9.265 | 0.026 |
| No - N (%) | 20 (52.6%) | 17 (89.5%) | 46 (61.3%) | 54 (71.1%) |  |  |
| **14_Sustainability of specific ingredients, foods, products or dietary patterns** | | | | | | |
| Yes -N (%) | 13 (34.2%) | 9 (47.4%) | 30 (40.0%) | 31 (40.8%) | 0.980 | 0.806 |
| No - N (%) | 25 (65.8%) | 10 (52.6%) | 45 (60.0%) | 45 (59.2%) |  |  |
| **15_Impact of adopting a SDP on social determinants (affordability, acceptability…)** | | | | | | |
| Yes -N (%) | 16 (42.1%) | 8 (42.1%) | 42 (56.0%) | 38 (50.0%) | 2.501 | 0.475 |
| No - N (%) | 22 (57.9%) | 11 (57.9%) | 33 (44.0%) | 38 (50.0%) |  |  |
| **16_Aspects affecting sustainable food habits (schools, medical stuff, parental support, public health experts, etc)** | | | | | | |
| Yes -N (%) | 18 (47.4%) | 10 (52.6%) | 44 (58.7%) | 39 (51.3%) | 1.528 | 0.676 |
| No - N (%) | 20 (52.6%) | 9 (47.4%) | 31 (41.3%) | 37 (48.7%) |  |  |
| **17_None of the above** | | | | | | |
| Yes -N (%) | 4 (10.5%) | 4 (21.1%) | 0 (0%) | 1 (1.3%) | 21.424 | <0.001 |
| No - N (%) | 34 (89.5%) | 15 (78.9%) | 75 (100%) | 75 (98.7%) |  |  |

| **TABLE S3.2** Informational gaps identified by dietitians depending on area of expertise | | | | |
| --- | --- | --- | --- | --- |
|  | **Clinical dietitian** | **Public Health Nutritionist** | **Chi-squared** | **p value** |
| **2_Lack of National/International legislation** | | | | |
| Yes -N (%) | 57 (50.0%) | 52 (56.5%) | 0.869 | 0.351 |
| No - N (%) | 57 (50.0%) | 40 (43.5%) |  |  |
| **3_Updated national food based dietary recommendations to include sustainability aspect** | | | | |
| Yes -N (%) | 66 (57.9%) | 55 (59.8%) | 0.075 | 0.784 |
| No - N (%) | 48 (42.1%) | 37 (40.2%) |  |  |
| **4_Knowledge (gap) needed to frame SDP in the global and national policy landscape** | | | | |
| Yes -N (%) | 51 (44.7%) | 49 (53.3%) | 1.481 | 0.224 |
| No - N (%) | 63 (55.3%) | 43 (46.7%) |  |  |
| **5_Lack of a common definition of sustainable diet/ dietary pattern** | | | | |
| Yes -N (%) | 60 (52.6%) | 47 (51.1%) | 0.049 | 0.825 |
| No - N (%) | 54 (47.4%) | 45 (48.9%) |  |  |
| **6_Information on product environmental footprint / sustainability on packaging (e.g. nutritional / environmental scores and sustainability labelling)** | | | | |
| Yes -N (%) | 63 (55.3%) | 58 (63.0%) | 1.272 | 0.259 |
| No - N (%) | 51 (44.7%) | 34 (37.0%) |  |  |
| **7_Sustainability literacy (Clear understanding of gas emissions, land/water use, biodiversity)** | | | | |
| Yes -N (%) | 53 (46.5%) | 64 (69.6%) | 11.047 | <0.001 |
| No - N (%) | 61 (53.5%) | 28 (30.4%) |  |  |
| **8_Sustainability of food processing** | | | | |
| Yes -N (%) | 60 (52.6%) | 52 (56.5%) | 0.311 | 0.577 |
| No - N (%) | 54 (47.4%) | 40 (43.5%) |  |  |
| **9_Sustainability of food packaging** | | | | |
| Yes -N (%) | 57 (50.0%) | 49 (53.3%) | 0.217 | 0.642 |
| No - N (%) | 57 (50.0%) | 43 (46.7%) |  |  |
| **10_Sustainability of food transportation** | | | | |
| Yes -N (%) | 57 (50.0%) | 46 (50.0%) | 0.000 | 1.000 |
| No - N (%) | 57 (50.0%) | 46 (50.0%) |  |  |
| **11_What aspects of the food systems contribute more or less to the environmental footprint (carbon emissions, land use, water use…)** | | | | |
| Yes -N (%) | 46 (40.4%) | 48 (52.2%) | 2.869 | 0.090 |
| No - N (%) | 68 (59.6%) | 44 (47.8%) |  |  |
| **12_Impact of food origin (location) on sustainability** | | | | |
| Yes -N (%) | 49 (43.0%) | 54 (58.7%) | 5.028 | 0.025 |
| No - N (%) | 65 (57.0%) | 38 (41.3%) |  |  |
| **13_Information on how to access local and seasonal food** | | | | |
| Yes -N (%) | 40 (35.1%) | 31 (33.7%) | 0.044 | 0.834 |
| No - N (%) | 74 (64.9%) | 61 (66.3%) |  |  |
| **14_Sustainability of specific ingredients, foods, products or dietary patterns** | | | | |
| Yes -N (%) | 44 (38.6%) | 38 (41.3%) | 0.156 | 0.693 |
| No - N (%) | 70 (61.4%) | 54 (58.7%) |  |  |
| **15_Impact of adopting a SDP on social determinants (affordability, acceptability…)** | | | | |
| Yes -N (%) | 52 (45.6%) | 51 (55.4%) | 1.964 | 0.161 |
| No - N (%) | 62 (54.4%) | 41 (44.6%) |  |  |
| **16_Aspects affecting sustainable food habits (schools, medical stuff, parental support, public health experts, etc)** | | | | |
| Yes -N (%) | 57 (50.0%) | 53 (57.6%) | 1.184 | 0.276 |
| No - N (%) | 57 (50.0%) | 39 (42.4%) |  |  |
| **17_None of the above** | | | | |
| Yes -N (%) | 5 (4.4%) | 3 (3.3%) | 0.173 | 0.678 |
| No - N (%) | 109 (95.6%) | 89 (96.7%) |  |  |
